# Icariin induces apoptosis by suppressing autophagy in tamoxifen-resistant breast cancer cell line MCF-7/TAM

**DOI:** 10.1007/s12282-019-00980-5

**Published:** 2019-06-06

**Authors:** Xia Cheng, Shirui Tan, Feifei Duan, Qingqing Yuan, Qingrong Li, Gang Deng

**Affiliations:** 1 School of Agriculture, Yunnan University, Kunming, 650500 Yunnan China; 2 College of Agriculture and Life Sciences, Kunming University, Kunming, 650241 Yunnan China; 3 The Second Affiliated Hospital of Kunming Medical University, Kunming, 650101 Yunnan China

**Keywords:** Breast cancer, Apoptosis, Autophagy, Drug resistance, Icariin

## Abstract

**Background:**

Icariin is a major component isolated from *Epimedium brevicornum Maxim* and has been reported to exhibit anti-tumor activity. However, whether icariin could reverse the acquired drug resistance in breast cancer remains largely unclear. Therefore, this study was designed to explore the antitumor effects of icariin and its underlying mechanisms in a tamoxifen-resistant breast cancer cell line MCF-7/TAM.

**Methods:**

3-(4,5-dimethylthiazol-2-yl)-2,5-diphenyltetrazolium bromide (MTT) assay and Lactate dehydrogenase (LDH) assay were performed to determine the effects of icariin on cell viability and cell death. Cell cycle progression and apoptosis were detected by flow cytometry analysis. Transmission electron microscopy (TEM) assay was utilized to observe cell autophagy. The downstream protein levels were measured using western blotting.

**Results:**

Here, we observed that icariin treatment not only inhibited the growth of MCF-7 but also has a potential function to overcome tamoxifen resistance in MCF-7/TAM. Moreover, icariin significantly induced cell cycle G0/G1 phase arrest and apoptosis, as well as suppressed autophagy. At molecular levels, icariin treatment remarkably down-regulated the expression levels of CDK2, CDK4, Cyclin D1, Bcl-2, LC3-1, LC3-II, AGT5, Beclin-1, but upregulated the expression levels of caspase-3, PARP and p62. Most importantly, we found inhibition of autophagy via 3-MA treatment could significantly enhance the effects of icariin on cell viability and apoptosis. Enhanced autophagy via autophagy related 5 (*ATG5*) overexpression could partially reverse the effects of icariin on cell viability and apoptosis.

**Conclusion:**

These results revealed that icariin might potentially be useful as an adjuvant agent in cancer chemotherapy to enhance the effect of tamoxifen through suppression of autophagy in vitro and provide insight into the therapeutic potential of icariin for the treatment of chemo-resistant breast cancer.

## Introduction

Breast cancer, the most common diagnosed cancer in women in the worldwide, is a heterogenous disease with multiple histological subtypes [1]. According to the American Cancer Society data, about 252,710 new cases and 40,610 deaths occurred in the US in 2017 [2]. Over 70% of all breast cancer patients are considered as estrogen receptor-positive (ER^+^), which contributes to tumorigenesis [3]. Tamoxifen (TAM) is an endocrine reagent with antioestrogenic effects, which has been the first clinically successful ER modulator (SERM) for all stages of ER^+^ breast cancer [4, 5]. Unfortunately, acquired resistance to tamoxifen limits its therapeutic effectiveness and results in rapid disease progression in breast cancer patients [6].

Programmed cell death (PCD), including apoptosis, autophagy, and programmed necrosis, can maintain homeostasis of the cell death with cell survival of normal cells and disturbance of equilibrium and PCD are critical in cancer cell fate determination [7]. Apoptosis (Type I PCD) is characterized as distinctive morphological and biochemical features, such as cell shrinkage and pyknosis, extensive plasma membrane blebbing and detachment of cell–cell adhesion [8]. The process of apoptosis can be triggered by different groups of executioner and regulatory molecules when exposure to a variety of physiological and pathological stimuli and conditions [8]. For the majority of malignancies, tumor cells must acquire the capacity of overcoming apoptosis to allow them survival [6].

Type II PCD is referred to as autophagy, known as garbage disposal and housekeeping functions [9, 10]. It begins with the formation of autophagosomes, which envelops part of the cytoplasm and delivers cytoplasmic components to the degradative organelle (lysosomes/vacuole) for breakdown and recycling [11, 12]. Autophagy was found to be an important regulator of a complex series of physiological processes, including cell survival, death, differentiation, and starvation [13]. Dysfunctional of autophagy is increasingly associated with cancer progression, neurodegeneration, inflammation, and infection [14]. Several studies have reported the induction of autophagy by chemotherapeutic drugs could reduce the apoptosis in cancer cells [15, 16].

Icariin (molecular formula: C_33_H_40_O_15_, molecular weight: 676.67 g/mol), a natural flavonoid glycoside that extracted from the traditional Chinese medical plant *Herba Epimedii* [17], has been found to possess anti-inflammatory, antioxidant, antidepressant and aphrodisiac effects [18, 19]. The most promising effect of icariin at cardiovascular level is the promotion of stem cell differentiation into beating cardiomyocytes, making it apply in cardiac cell therapy [20, 21]. In addition, icariin displays pharmacologically active effects on rheumatoid arthritis [22], live disease [23], diabetic nephropathy [24], and even on cancer [25]. Recently, emerging studies have reported icariin regulates cell proliferation, apoptosis and autophagy in various tumors. For example, Ren et al. showed that icariin inhibited osteosarcoma cell proliferation [26]. Similarly, icariin exerted suppressive effects on colon cancer cells [27], thyroid cancer cells [28] and ovarian cancer cells [29]. The induction of S-phase arrest and apoptosis were observed in medulloblastoma cells after treatment with icariin [30]. Interestingly, Jiang et al. demonstrated that icariin significantly enhanced the chemosensitivity of cisplatin-resistant ovarian cancer cells by suppressing autophagy [31]. Moreover, icariin could effectively attenuate paclitaxel-induced neuropathic pain [32] and chemotherapy-induced bone marrow microvascular damage [33]. Based on these evidences, we thus speculated that icariin might play an important role in TAM resistance.

In this study, we aimed to investigate the biological function of icariin in TAM resistance in breast cancer cells by presenting some evidences regarding the activity of icariin on viability, LDH cytotoxicity, cell cycle progression, apoptosis, and autophagy of MCF-7/TAM cells. We also investigated the role of icariin in the molecular mechanism underlying the reversal of TAM resistance in breast cancer cells. The present study might shed new light on reversing drug resistance and providing a reference for clinical applications.

## Materials and methods

### Cell culture and drug treatment

Human breast cancer cell lines, MCF-7, T47D and the corresponding TAM-resistant cell lines (MCF-7/TAM and T47D/TAM) were obtained from Cell Bank of the Chinese Academy of Sciences (Shanghai, China) and cultured in Dulbecco’s Modified Eagle’s Media (DMEM) medium with 10% PBS. To maintain TAM resistance, MCF-7/TAM and T47D/TAM cells were continuously cultured in a medium containing additional 3 μmol/L TAM (Sigma-Aldrich) for at least 6 months. Cell cultures were maintained a humidified atmosphere containing 5% CO_2_ at 37 °C. In the in vitro experiments, MCF-7/TAM cells were divided into four groups according to the following treatments: (1) no drug in the control (blank) group; (2) Icariin (10, 25, 50 and 75 μM) group; (3) 3-methyladenine (3-MA) (2.5 mM, Sigma-Aldrich) group; (4) Combination (3-MA + Icariin) group.

### Plasmids and transfection

The cDNA sequence of *ATG5* was cloned into pcDNA3.1 expression vector to construct recombinant pcDNA3.1-*ATG5* vector by Sangon Biotech Co. Ltd. (Shanghai, China) and confirmed by gene sequencing. In addition, pcDNA3.1 vector was used as the negative control (NC). For cell transfection, MCF-7/TAM cells in Icariin group at a density of 2 × 10^5^ cells per well were grown in six-well plates and transfected with pcDNA3.1-*ATG5* or NC using Lipofectamine 2000 according to the manufacturer’s instructions (Invitrogen, USA).

### MTT assay

Cell viability was determined using MTT assay in breast cancer cells. In brief, cells were seeded at density of 1 × 10^4^/well into 96-well plates and incubated at 37 °C for 24 h under 5% CO_2_ at 37 °C. After different treatments, 20 μL of MTT solution (5 mg/ml) was added into each well and each plate was further incubated for 4 h at 37 °C. The generated formazan in individual wells was dissolved in 200 μL DMSO and the absorbance was measured at 570 nm using a microplate reader (Epoch, Bio-Tek, VT, USA). The cell viability was expressed as percentage inhibition relative to controls. The half-maximal inhibitory concentration (IC_50_) was calculated from the dose–response curve using Origin 8.0 software (Origin Lab Corporation, Northampton, MA, USA).

### Lactate dehydrogenase (LDH) assay

Cell injury was evaluated based on LDH leakage into the culture medium from cells using an LDH assay kit (Sigma-Aldrich) according to the manufacturer’s instruction. The amount of LDH was determined by measuring the optical density at 450 nm.

### Flow cytometry analysis

The cell cycle distribution of MCF-7/TAM cells was estimated using a flow cytometer by quantitation of DNA content of cells stained with PI. In brief, MCF-7/TAM cells were seeded on 6-cm dishes and harvested until 80% confluence. Then cells were fixed overnight at 4 °C with 70% ethanol, followed by resuspension in 500 μL of PBS. Subsequently, the pellets were incubated in PBS containing PI and RNase (10 mg/mL) for at least 30 min at 37 °C. Afterwards, cellular DNA content was analyzed on a flow cytometer (BD Biosciences, San Jose, CA).

Cell apoptosis was detected using Annexin V-APC/7-AAD apoptosis detection kit (KeyGEN Biotech, China) and analyzed by flow cytometry (Becton Dickson, USA). The early (Annexin V +/7-AAD-) and late apoptotic (Annexin V +/7-AAD +) cells were quantitated, respectively.

### Transmission electron microscopy (TEM)

MCF-7/TAM cells were cultured in the presence of media or icariin for 24 h. Then cells were harvested and fixed overnight at 4 °C in 2.5% glutaraldehyde and rinsed with 0.1 M cacodylate buffer. Subsequently, cells were then posted-fixed in 1% osmium tetroxide for 2 h at 4 °C, dehydrated in a graded series of ethyl alcohol, and embedded in epoxy resin. The ultrastructures of cells undergoing autophagy were examined under a Philips CM120 transmission electron microscope (Eindhoven, The Netherlands).

### Western blot analysis

Total proteins were extracted using RIPA agents (Beyotime, China) and the concentration of the proteins was detected by BCA Protein Assay kit. The protein extracts were separated on 5–15% sodium dodecyl sulfate (SDS)-PAGE and then transferred to a PVDF membrane (Millipore, USA). After blocked with 5% nonfat milk for 1 h at room temperature, the membranes were incubated with primary antibodies against CDK2 (1:1000, #2546, Cell signaling), CDK4 (1:1000, 11026-1-AP, Proteintech), Cyclin D1 (1:1000, 60186-1-1 g, Proteintech), Bcl-2 (1:1000, #2876, Cell signaling), Caspase-3 (1:500, #9661, Cell signaling), PARP (1:1000, #9542, Cell signaling), LC3 (1:1000, #7851, Cell signaling), ATG5 (1:2000, 12036-1-AP, Proteintech), p62 (1:500, #1354, Cell signaling), Beclin-1 (1:1000, #4578, Cell signaling) and GAPDH (1:10000, 10494-1-AP, Proteintech) at 4 °C overnight. After washing with PBS, the membranes were incubated with horseradish peroxidase-conjugated secondary antibody for 2 h at room temperature. The blots were detected using enhanced chemiluminescence detection kit (Beyotime Institute of Biotechnology). GAPDH was used as an internal control.

### Statistical analysis

All quantitative data were expressed as mean ± standard deviation (SD) of three independent experiments. All statistical parameters were calculated with GraphPad Prism 6.01 (GraphPad Software Inc.). Student’s *t* test was used to analyze the difference between two groups. The differences of multiple groups were calculated by one-way ANOVA with post hoc test. Differences were considered statistically significant at *p* < 0.05.

## Results

### The effects of icariin on the viability of TAM-resistant breast cancer cell lines

**Fig. 1:**
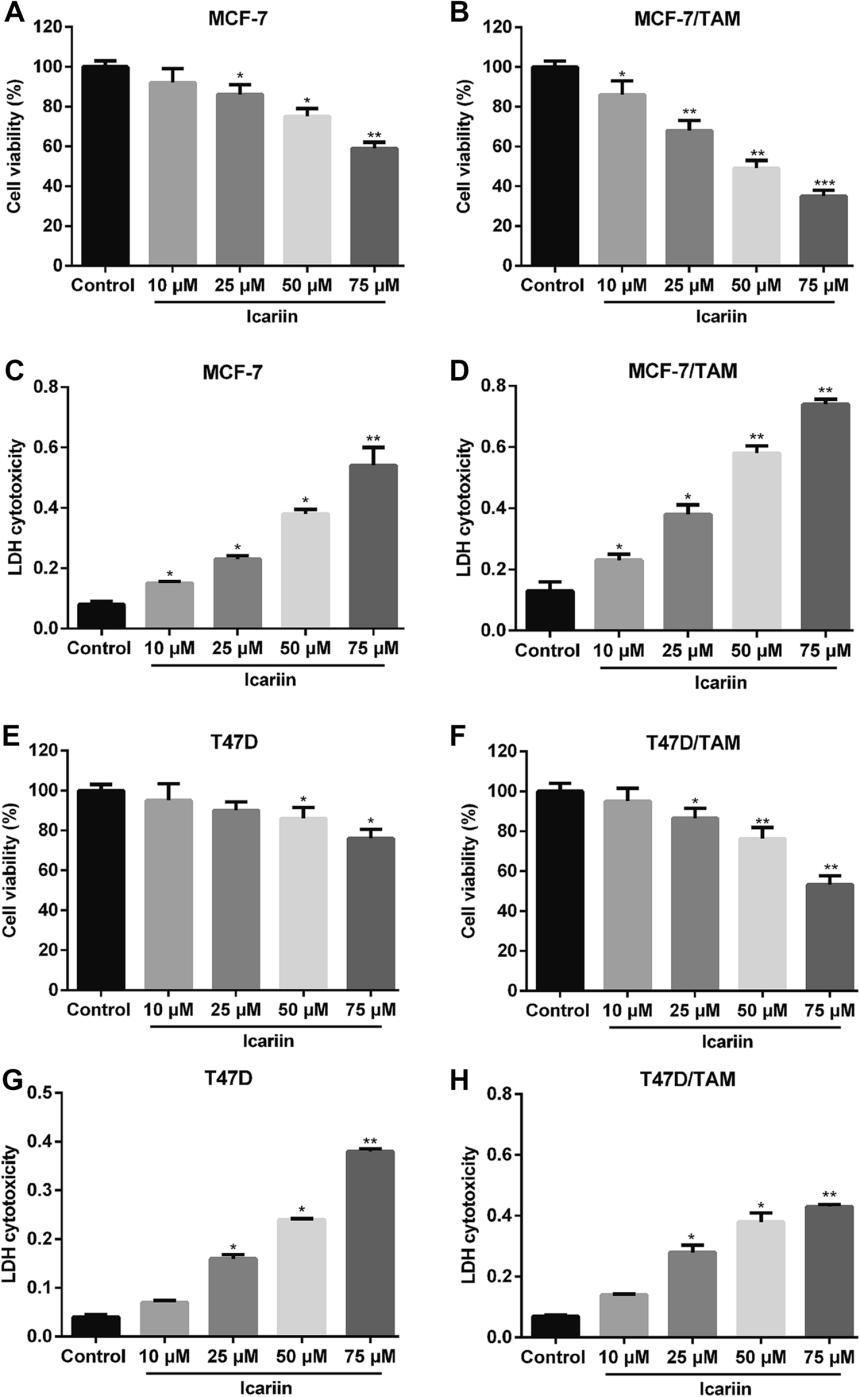
Growth inhibition of tamoxifen (TAM)-sensitive and resistant breast cancer cell lines by icarrin treatment in a dose-dependent manner. Cells were treated with the indicated concentrations (10 μM, 25 μM, 50 and 75 μM) of icarrin for 24 h, and cell viability was determined in **a** MCF-7, **b** MCF-7/TAM, **c** T47D and **d** T47D/TAM cells by 3-(4,5-dimethylthiazol-2-yl)-2,5-diphenyltetrazolium bromide (MTT). The LDH activity was measured in **e** MCF-7, **f** MCF-7/TAM, **g** T47D and **h** T47D/TAM cells using Lactate dehydrogenase (LDH) assay. Data represent the mean ± SD of three independent experiments (*n* = 3). *p < 0.05, **p < 0.01, ***p < 0.001, as compared with blank control

To investigate the cytotoxic effect of icariin in breast cancer cells, two TAM-sensitive breast cancer cell lines and the corresponding TMA-resistant cell lines were treated with icariin at increasing concentrations for 24 h. Using MTT assay, we observed that icariin showed dose-dependent anti-proliferative activity in TAM-sensitive MCF-7 (Fig. 1a, *p* < 0.05, *p* < 0.01) and resistant MCF-7/TAM cells (Fig. 1b, *p* < 0.05, *p* < 0.01, *p* < 0.001). Similarly, the cell viability of T47D (Fig. 1c, *p* < 0.05) and T47D/TAM (Fig. 1d, *p* < 0.05, *p* < 0.01) cells was significantly decreased after icariin treatment in a dose-dependent manner. The IC50 value of icariin for MCF-7, T47D and T47D/TMA was more than 75 μM, while IC50 value for MCF-7/TAM cells was approximately 50 μM. In addition, icariin treatment caused increased LDH activity in these four cell lines in a dose-dependent manner. Consistently, increased LDH activity was more obvious in MCF-7/TAM cells compared with the other three cell lines (Fig. 1e–h, *p* < 0.05, *p* < 0.01). These preliminary results suggest that icariin exerts anti-proliferation activity in breast cancer cells. Notably, MCF-7/TAM cells were more sensitive to icariin compared with the other three cell lines, which were thus selected for the further in vitro experiments.

### The effects of icariin on the cell cycle progression and apoptosis of the drug-resistant MCF-7/TAM cell line

**Fig. 2:**
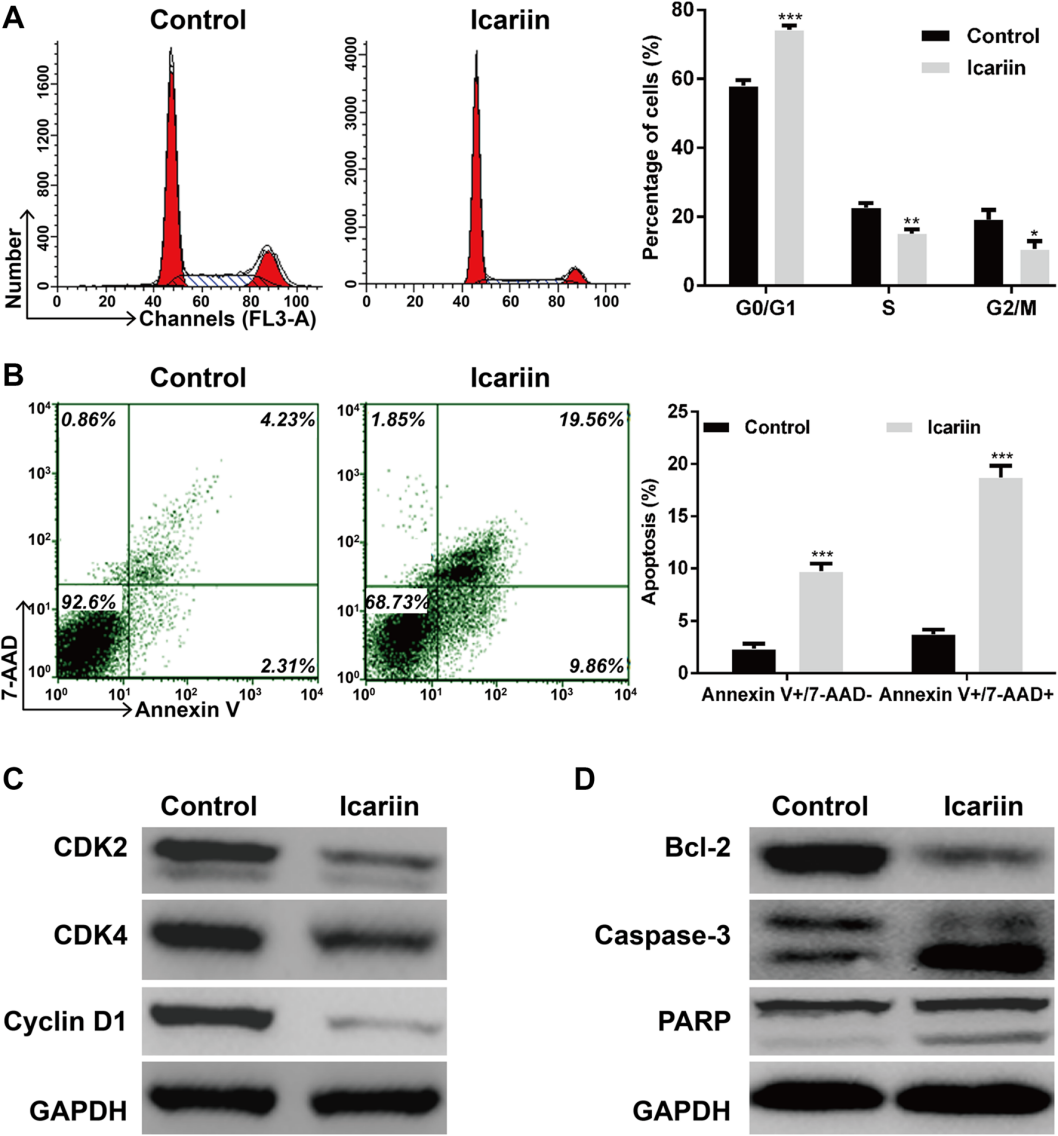
The effects of icarrin on the cell cycle progression and apoptosis in MCF-7/TAM cells. **a** Flow cytometry combined with PI staining was used to analyze cell cycle distribution in MCF-7/TAM cells after icariin treatment. **b** Flow cytometry combined with Annexin V plus 7-AAD staining was used to analyze cell apoptosis in MCF-7/TAM cells after icariin treatment. Data represent the mean ± SD of three independent experiments (*n* = 3). *p < 0.05, **p < 0.01, ***p < 0.001, as compared with blank control; Western blotting analysis of **c** cell cycle regulators, including cyclin-dependent kinase 2 (CDK2), CDK4 and Cyclin D1) and **d** apoptotic markers, including (cleaved caspase-3), poly ADP ribose polymerase (PARP) and B-cell lymphoma 2 (Bcl-2) in MCF-7/TAM cells after icariin treatment

Based on the IC_50_ of icariin concentration, we further investigated the mechanisms underlying the icariin-mediated reversal of drug resistance by analyzing the effect of icariin on cell cycle progression and apoptosis using flow cytometry in MCF-7/TAM cells. As shown in Fig. 2a, the cell percentage of G0/G1 phase (58.06 ± 1.58% vs. 74.22 ± 1.28%, *p* < 0.001) in icariin group was significantly increased, while the cell percentage of S (22.73 ± 1.28% vs. 15.17 ± 1.12%, *p* < 0.01) and G2/M (19.21 ± 2.82% vs. 10.61 ± 2.37%, *p* < 0.05) phase was decreased, as compared with control group in MCF-7/TAM cells. In addition, icariin treatment promoted more early and late apoptosis compared with control cells. In total, an approximately fivefold increase in apoptotic populations in icariin group compared to control group in MCF-7/TAM cells (Fig. 2b).

Furthermore, the expression alterations of some cell cycle regulators and apoptotic markers were detected using western blotting. Our results showed that the expression of CDK2, CDK4 and Cyclin D1, associated with G1-S transition, were decreased in icariin group (Fig. 2c). Besides, the expression levels of cleaved caspase-3 and PARP were increased, while the anti-apoptotic protein Bcl-2 was downregulated in icariin group compared with control group (Fig. 2d).

### Inhibition of icariin induced cell autophagy could enhance the apoptotic effects of icariin in MCF-7/TAM cells

**Fig. 3:**
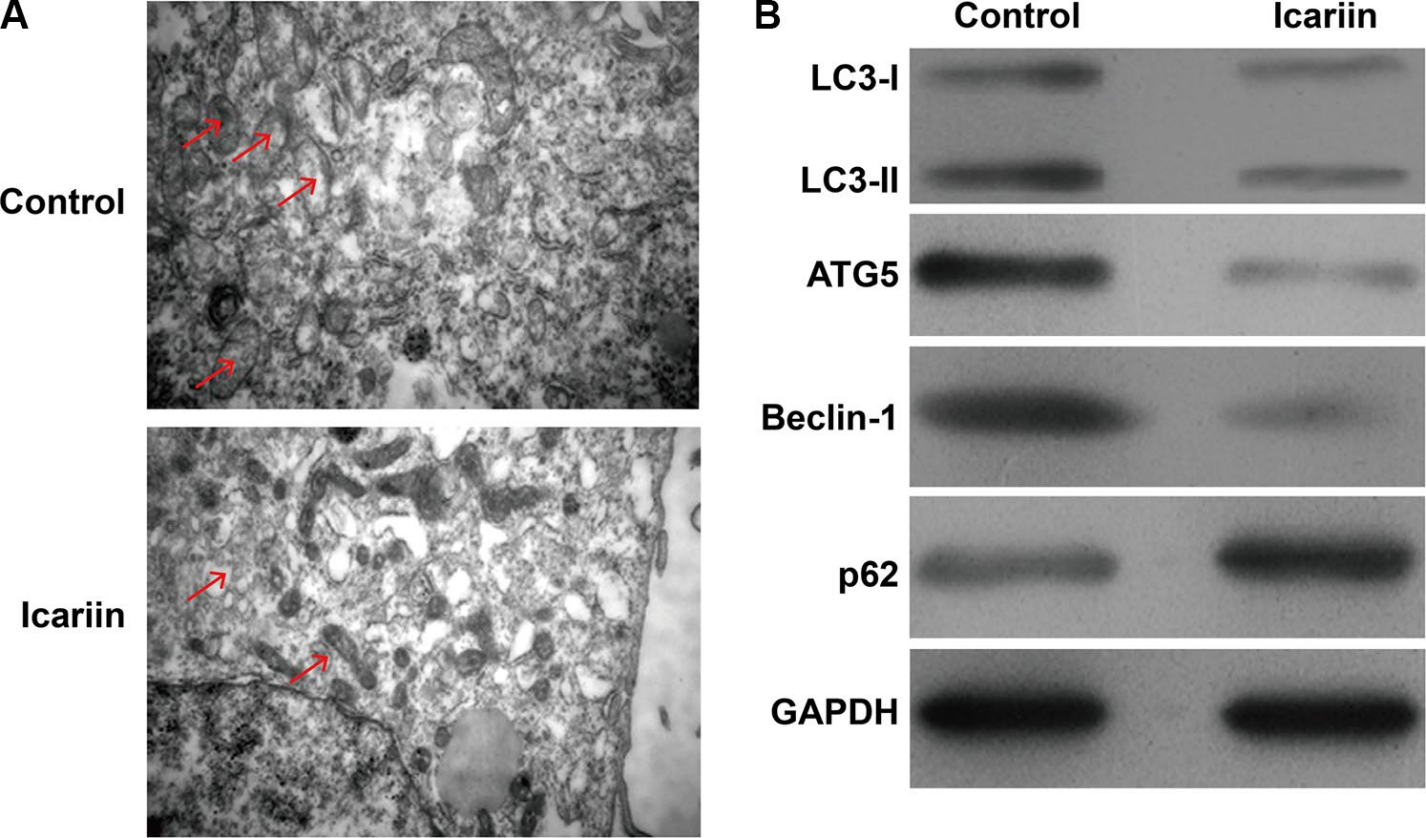
Effects of icarrin treatment on autophagy inhibition in MCF-7/TAM cells. **a** Transmission electron microscope observation of cell autophagosomes. **b** The protein levels of various autophagy markers were analyzed by western blot in MCF-7/TAM cells after treatment with icariin. GAPDH was used as an internal control

Accumulating evidence have shown that autophagy may act as a protective response in protecting tumor cells from chemotherapeutic agents-induced cell apoptosis, which is a major problem for targeted chemotherapies. Here, we first assessed whether icariin affected autophagy in MCF-7/TAM cells. As shown in Fig. 3a, icariin treatment caused reduced autophagic vacuoles compared with control cells. We then determined the effects of icariin on the expression of proteins involved in the autophagy pathway using western blot analysis. As expected, icariin treatment markedly suppressed the conversion of microtubule-associated protein light chain (LC3)-I to LC3-II, decreased the expression of autophagy makers, ATG5 and Beclin-1. Consistently, autophagy inhibition in icariin-treated cells caused p62 accumulation (Fig. 3b).

**Fig. 4:**
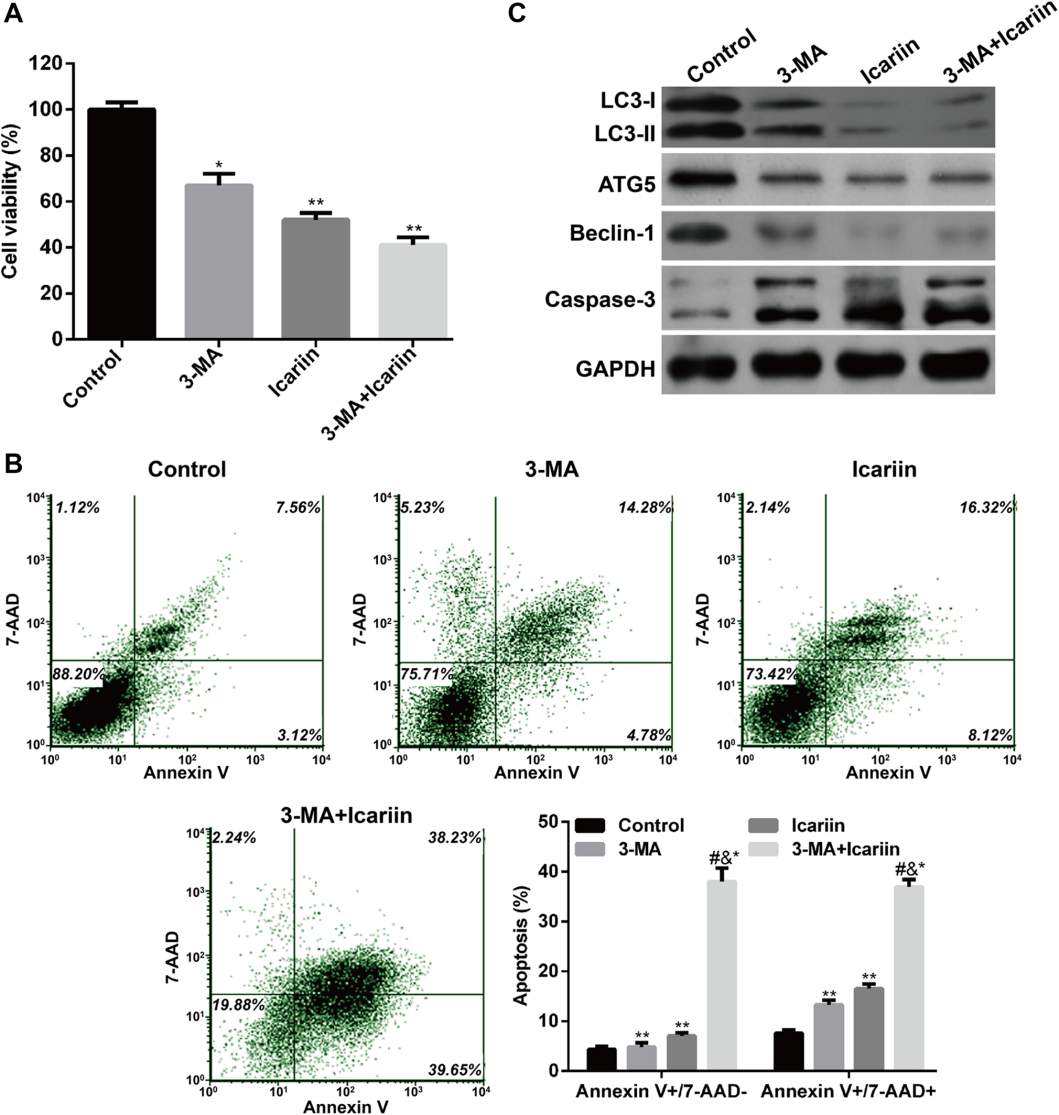
Icarrin treatment reverses the drug resistance of MCF-7/TAM cells by reducing autophagy. Cells were treated with 2.5 mM 3-MA and/or 50 μM icariin for 24 h. **a** The effects of 3-MA-mediated autophagy inhibition on icariin induced cell death were detected by MTT assay. **b** Flow cytometry combined with Annexin V plus 7-AAD staining was used to analyze cell apoptosis. Data represent the mean ± SD of three independent experiments (*n* = 3). *p < 0.05, **p < 0.01, as compared with blank control; ^#^*p* < 0.05, as compared with 3-MA; ^&^*p* < 0.05, as compared with icariin treatment; **c** The protein levels of various autophagy and apoptotic markers were analyzed by western blot. GAPDH was used as an internal control

To further confirm whether inhibition of autophagy is involved in icariin-induced apoptosis, an autophagy inhibitor, 3-methyladenine (3-MA) was used to pretreat MCF-7/TAM cells with or without icariin treatment. MTT assay showed the cell viability was significantly decreased in MCF-7/TAM cells treated with solely 3-MA or icariin or combination with 3-MA and icariin, in comparison with control cells. Notably, combination treatment with 3-MA and icariin presented the most suppressive effects in cell viability, as compared with MCF-7/TAM cells treated with either 3-MA or icariin (Fig. 4a, *p* < 0.05, *p* < 0.01). Furthermore, co-treatment of MCF-7/TAM cells with 3-MA and icariin significantly promoted early and late apoptosis, as compared to treatment with either 3-MA or icariin (Fig. 4b, *p* < 0.05). In molecular levels, co-treatment of MCF-7/TAM cells with 3-MA and icariin significantly suppressed LC3-I to LC3-II conversion, ATG5 and Beclin-1 expression, and increased the cleavage of caspase-3, as compared to treatment with either 3-MA or icariin (Fig. 4c). These results indicated that icariin could effectively inhibit autophagy in MCF-7/TAM cells, which might be implicated in icariin-mediated induction of apoptosis.

### Enhanced autophagy by ATG5 overexpression could partially reverse enhanced apoptosis by icariin treatment

**Fig. 5:**
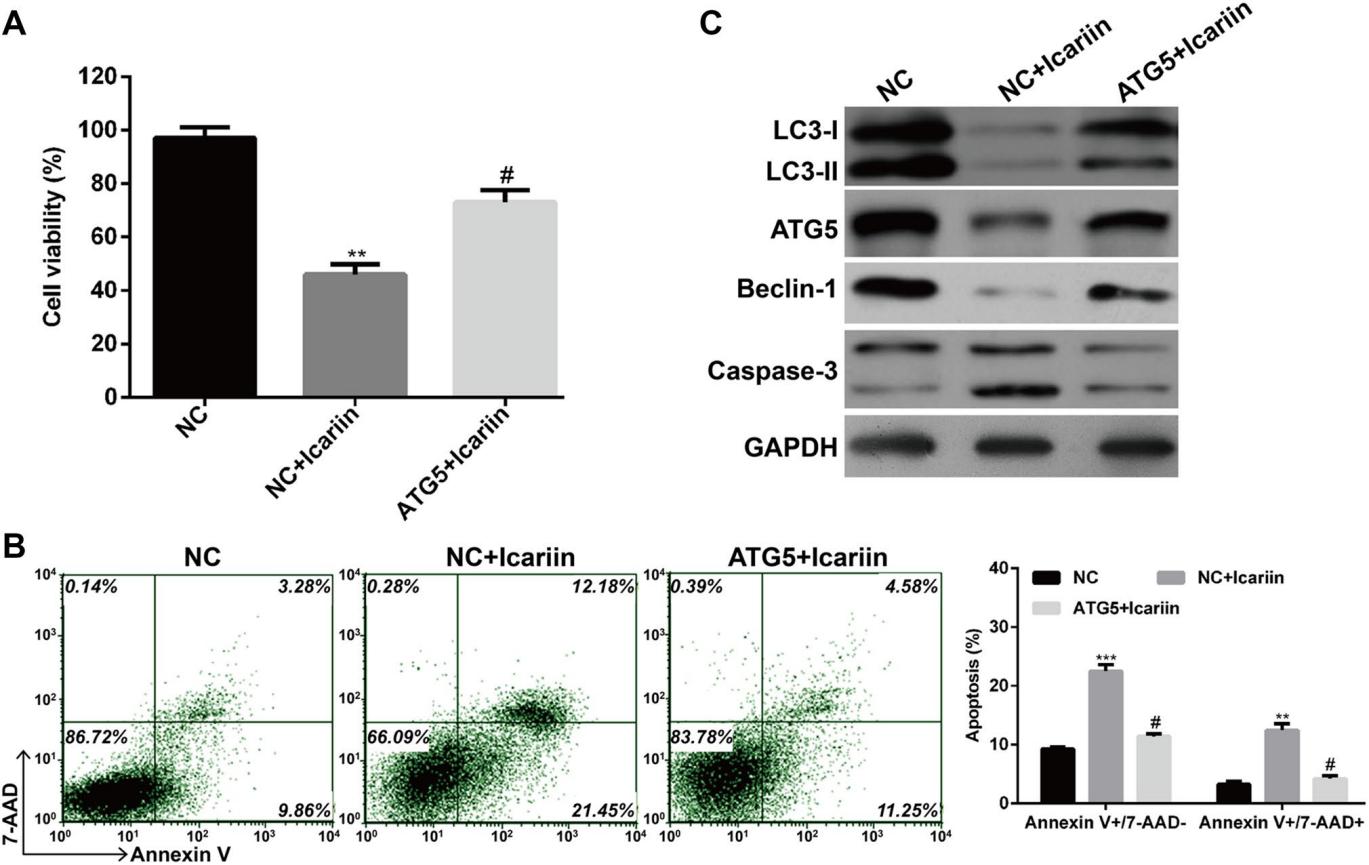
Enhanced autophagy by *ATG5* overexpression could partially reversed enhanced apoptosis by icarrin treatment. MCF-7/TAM cells were transfected with pcDNA3.1-*ATG5* or empty pcDNA3.1 vector as negative control (NC), and then treated with icariin. **a** Cell viability was determined by MTT assay. **b** Flow cytometry combined with Annexin V plus 7-AAD staining was used to analyze cell apoptosis. Data represent the mean ± SD of three independent experiments (*n* = 3). **p < 0.01, ***p < 0.001, as compared with NC; ^#^*p* < 0.05, as compared with NC + icariin; **c** The protein levels of various autophagy and apoptotic markers were analyzed by western blot. GAPDH was used as an internal control

To further investigate whether icariin induced apoptosis correlated with autophagy, the expression of *ATG5*, a well-established autophagy-related gene, was overexpressed in MCF-7/TAM cells after treatment with icariin. We first found upregulation of *ATG5* could partially reverse the inhibition of cell viability induced by icariin treatment in MCF-7/TAM cells (Fig. 5a, *p* < 0.05, *p* < 0.01). Consistently, the elevated apoptotic rate of MCF-7/TAM cells after icariin treatment was significantly alleviated by *ATG5* overexpression (Fig. 5b, *p* < 0.05, *p* < 0.01, *p* < 0.001). Subsequently, the expression of autophagy and apoptosis-related protein was determined by western blotting. As shown in Fig. 5c, the expression levels of LC3-I to LC3-II conversion, Beclin-1 and *ATG5* were markedly increased, but caspase-3 was decreased after *ATG5* overexpression. Collectively, these results strongly supported that autophagy inhibition by icariin treatment contributed to icariin-induced apoptosis.

## Discussion

The tumor microenvironment is a unique biological environment that promotes tumorigenesis, tumor metastasis and therapeutic resistance, including breast cancer [34]. TAM, as a selective estrogen receptor (ER) modulator, has been used as the first-line treatment for ER-positive breast cancer for many years, but its effectiveness is limited as most advanced breast cancer eventually recur with acquired resistance despite initial responsiveness to TAM [35]. It is estimated approximately 40% of breast cancer patients relapse with acquired endocrine resistant disease progression [36]. Therefore, altering the tumor microenvironment to improve therapeutic TAM resistance is urgently needed for breast cancer treatment. Recently, icariin was found to significantly enhance the chemosensitivity of cisplatin-resistant ovarian cancer cells by suppressing autophagy [31]. Moreover, icariin exerts anti-tumor effects on several tumor cells, including gallbladder cancer [37], ovarian cancer [38], colorectal cancer [39], and esophageal cancer [40]. However, whether icariin could promote the chemosensitivity of TAM-resistant breast cancer cells remains unclear.

The current study selected two TAM-resistant breast cancer cell lines to investigate the effects of icariin on them. Our results showed MCF-7/TAM cells were more sensitive to icariin compared with T47D/TAM, which were thus selected for further in vitro experiments. Previous reports demonstrated that icariin could block cell cycle progression in the G0/G1 phase in human osteosarcoma cells and mouse melanoma B16 cells [30]. Similarly, we found that icariin could induce G0/G1 phase arrest in MCF-7/TAM cells. Eukaryotic cell cycle deregulation has a strong link with carcinogenesis, which is regulated by cyclins and cyclin-dependent kinases (CDKs) [41]. Deregulation of CDK/cyclin complex activity is observed in a variety of human tumors [42]. In our results, we found that icariin significantly down-regulated the expression of CDK2, CDK4, and Cyclin D1 in MCF-7/TAM cells. Herein, we suggested that icariin could cause G0/G1 arrest in MCF-7/TAM cells.

In addition, there was a significantly elevated apoptosis in MCF-7/TAM cells after icariin treatment as compared to controls. We further analyzed the protein expressions of Bcl-2, caspase-3, and PARP. It is well established that caspase-3 is a frequently activated death protease for the execution of apoptosis [43]. As a major death substrate of caspase 3, 6, and 7, cleavage of PARP is a convenient marker of apoptosis [44]. The anti-apoptotic Bcl-2 encodes an integral membrane protein that usually localizes on the outer membrane of mitochondria, and prevents apoptosis in most types of cells [45]. Our current study clearly demonstrated that treatment of MCF-7/TAM with icariin led to increased cleavage of caspase-3 and PARP, as well as decreased expression of Bcl-2. These observations suggest that icariin could promote cell apoptosis in MCF-7/TAM cells.

Furthermore, transmission electron microscope observations revealed that icariin treatment caused reduced autophagic vacuoles compared with control cells. During autophagosome formation, the gene product *ATG5* is required, and LC3-I is converted to LC3-II, which is a key maker of autophagy [46]. Beclin 1 is a mammalian autophagy protein involved in diverse biological processes, including tumor suppression and cell death [47]. P62 is an autophagy receptor that can be selectively degraded by autophagy [48]. In icariin-treated MCF-7/TAM cells, decreased autophagy was confirmed by decreased ATG5, Beclin 1, and conversion of LC3-I to LC3-II, along with increased p62.

Being an intracellular lysosomal degradation pathway of cellular components, autophagy was primarily found to allow cell survival [49]. Recently, autophagy is presented as a dual-function event for either promote cell survival or cell death [50]. Here, an autophagy inhibitor 3-MA could alleviate the cell apoptosis in MCF-7/TAM cells caused by icariin treatment. Conversely, *ATG5* overexpression exhibited the opposite effect. Therefore, it is likely that icariin may promote apoptosis partially through inhibition of autophagy in MCF-7/TAM cells.

## Conclusions

Taken together, we concluded that icariin could reverse TAM resistance in breast cancer cell line MCF-7 by inhibiting autophagy. In addition, some limitations that are presented in this study such as these results are needed to be repeated in multiple TAM-resistant breast cancer cell lines, and lack of in vivo animal experiments, which will be investigated in further research. To some degree, these preliminary results may hold great promise in improving the clinical outcomes of TAM-resistant breast cancer patients.
